# Telerehabilitation: A Practical Remote Alternative for Coaching and Monitoring Physical Kinetic Therapy in Patients with Mild and Moderate Disabling Parkinson's Disease during the COVID-19 Pandemic

**DOI:** 10.1155/2022/4370712

**Published:** 2022-08-08

**Authors:** Aurelian Anghelescu

**Affiliations:** ^1^“Carol Davila” University of Medicine and Pharmacy, Bucharest, Romania; ^2^Neurorehabilitation Clinic, Teaching Emergency Hospital “Bagdasar-Arseni”, Berceni Av., No. 12, Postal Code: 041915, 4th Sector, Bucharest, Romania

## Abstract

**Background:**

The COVID-19 pandemic imposed social/physical distancing, lockdown measures, and forced reorientation of the rehabilitation programs for people with Parkinson's disease (PD). Epidemiologic safety measures boosted remote exercise-based treatment.

**Objectives:**

Remote delivery of rehabilitation care services is not typically used in our department. Therefore, this study aimed to assess and implement a telehealth physical rehabilitation program tailored for outpatients with idiopathic PD and slight or medium functional limitations.

**Methods:**

A prospective study was performed on a group of outpatients with idiopathic PD, selected from the database of the neurorehabilitation clinic of the Emergency Teaching Hospital “Bagdasar-Arseni.” We studied 17 patients (5 women and 12 men), aged between 54-70 years (average 65.9 ± 4.87), with a disease history of 7.3 ± 3.6 (years), with mild or moderate disabling clinical forms, quantified by an average Hoehn and Yahr score of 2.3 ± 0.35 (limits 1.5-3). All patients underwent pharmacologic treatment with unchanged doses throughout the study. No patients had disabling osteoarticular problems (all could walk independently) and had no significant psycho-cognitive dysfunction. Patients were supervised and coached online in tandem by the therapist and physician. In addition, a family member assisted and supervised the patient's performance and coordinated the technical electronic procedures. Walking biodynamics was assessed by timing “6-meters walking” and “Get up and walk 3 meters” (TUG) tests. Each person attended ten sessions of motor telerehabilitation procedures (2 per week) lasting 50 minutes each during social distancing (October-December 2021).

**Results:**

None of the patients was at increased risk of falling. They all improved their locomotor performance, reflected in a significant decrease in TUG duration (the initial average time improved from 13.50 seconds to 10.57). The telerehabilitation program also significantly improved the average walking speed (initially, 44.5 cm/sec and finally, it raised to 56.8 cm/sec). *Discussion*. The TUG and “6-meters walking” tests are helpful tools for a global biodynamic remote assessment of PD patients. Limitations of the study: a small group of selected patients, restrictive working conditions (due to epidemiological social/physical restrictions and no direct physiotherapist-patient contact), and need for supervision by an attendant to assist the subject and perform the audio-video transmission. Further studies are necessary to identify the optimal web-based model of care and boost the implementation of this modern neurorehabilitation concept.

**Conclusions:**

Telemedicine turned the virtual space into a new reality and may compensate for the restrictions imposed on face-to-face meetings in pandemic conditions. Moreover, with modern telecommunication techniques, a regular and individualized physical kinetic rehabilitation program can be performed even in pandemic conditions. Remote delivery of kinetic motor programs was appropriate for selected groups of PD patients.

## 1. Background

During the last two and a half years, the successive pandemic waves gradually affected more than 521,920,560 people worldwide [1]. Nowadays, COVID-19 cases are rising again, and the newest Omicron subvariants are spreading quickly. During the pandemic era, a threatening question has arisen (as Damocles' sward), referring to the post-SARS-CoV-2 immune-mediated reactions as triggers for *α*-synucleinopathies [2] and neurodegeneration, including new cases of PD [3].

Parkinson's disease (PD) is the second most common neurodegenerative disease. It is a chronic, slowly progressive disease, clinically characterized by motor features (rigidity, bradykinesia, postural instability, and resting tremor) and non-motor issues (autonomic dysfunction, cognitive/neurobehavioral disturbances, sleep disorders, and sensory abnormalities, such as olfactory dysfunction, paresthesia, and pain). PD causes a continuously deteriorating quality of life and often leads to a significant caregiver burden [4–6].

PD has a decade-long disease course with evolving symptoms. These patients require tailored and highly specialized therapeutic management, regular care, periodic medical consultations, and drug adjustments. Patients with PD are a vulnerable population group and need a multidisciplinary, integrated approach to neurorehabilitation.

Nondrug therapy consists of kinetic physiotherapy and occupational therapy, speech and deglutition rehabilitation, neuropsychologic support, and nursing—which should be carried out frequently and continuously for the entire life.

As motor and cognitive disabilities progress, patients may be unable to travel long distances for regular follow-up visits and rehabilitation at tertiary medical centers [7, 8].

The coronavirus pandemic evolution and duration had severe repercussions on PD patients. Advanced age (mainly 75-79 years) and male PD were significantly predisposed to COVID-19 infection, with an overall post-infection mortality rate significantly higher than in non-PD patients (35.4% vs. 20.7%) [9]. An advanced, disabling clinical stage of PD, longer disease duration, and comorbidities were also associated with a higher COVID-19 mortality rate.

A comprehensive German database of 64,434 PD population revealed a dramatic decline (by up to 72.7%) in the number of in-hospital admissions in 2020 [9].

PD survivors after COVID-19 infection reported a significant and acute clinical worsening in either motor disturbances or neuropsychiatric non-motor symptoms [10, 11].

Overweight and obesity have reached pandemic proportions (“co-vesity”—a new pandemic within the COVID-19 era) [12]. Sedentarism and social isolation were significantly related to worse health outcomes and had pejorative repercussions on the quality of life. They accentuated the deficits in mobility, with consecutive a greater risk of falls. They are associated with overweight (as a consequence of prolonged immobility), dysfunctions of cognitive processing (memory and concentration), and communication issues (difficulty with speech) [13, 14].

Due to lockdown, outpatient rehabilitation services were suddenly interrupted [11, 15, 16]. Discontinuance of rehabilitative treatment was reported by 61% of PD patients [11], with negative repercussions in performing functional activities: deterioration in walking ability (in 37% of PD people), increased need for assistance (for 24.8%), and increased psychological stress (depression and anxiety), in 42% subjects.

To overcome the public health crises and the negative impact of social and mobility restrictions during COVID-19 lockdown constraints, the implementation of telehealth services in clinical practice offered promising areas: telemedicine, remote-/teleconsultation, and treatment (teleneurorehabilitation), and telemonitoring.

The Health Resources and Services Administration (HRSA) of the U.S. Department of Health and Human Services defines telehealth as “the use of electronic information and telecommunications technologies to support and promote long-distance clinical health care, patient and professional health-related education, public health and health administration” [17].

According to the current EU legislation, “Telemedicine is both a health service and an information society service” [18].

Telemedicine is particularly suited to evaluate patients with PD or other movement disorders of various etiopathology.

Information and communication technologies (ICTs) might represent a convenient way to provide accessible, cost-effective, and high-quality remote healthcare services in both developed and developing countries. The telehealth model promotes epidemiological safe remote medical services and delivers noncontact kinesiotherapy interventions. It might be used as a first-line platform for physical activity coaching programs for people with PD, in remote geographic zones, and during special conditions [19, 20].

The patients' and physicians' perceptions and satisfaction with telemedicine health services are high, no matter the health system setting (hospitals, community clinics, and long-term care facilities) [21–27].

The telehealth model encountered challenging limitations and barriers [27–29]:

- biological and psychological ones (linked to the patient's health status and disability, the risk for complications, his/her cognitive status/reserves, the educational level and difficulties in using communication technologies, lack of compliance and adherence to the physical program, depression, and anxiety)
- acceptance by the medical staff (the telehealth providers) of the up-to-date smart technologies or limited computer skills
- technology-related issues linked to the technical resources and their function, such as limited access or absence of electronic devices at home, lack of high-speed Internet and performant equipment/devices, and lack of technical assistance
- legislative ones (patients' privacy data)
- financial ones linked to the reimbursement of healthcare services
- limited possibilities for remotely correct examination (rigidity and balance evaluations may be challenging via telemedicine).

Remote delivery of rehabilitation care services is not typically used in our clinical neurorehabilitation department. Therefore, this prospective clinical study aimed to implement and assess the outcomes of a telehealth physical rehabilitation program guided by a therapist and physician, tailored for our chronic idiopathic PD subjects.

## 2. Material and Methods

The kinetic telerehabilitation program was proposed to 17 subjects selected from the outpatients of the Neurorehabilitation Clinic database who previously received motor rehabilitation, interrupted by the pandemic.

Written informed consent was obtained from each patient for the publication of any potentially identifiable images or data. The Bioethics Commission of the Teaching Emergency Clinical Hospital “Bagdasar-Arseni” approved the study.

The patient's selection criteria for an appropriate remote kinetic motor rehabilitation program are synthesized in Table 1. No patients had debilitating osteoarticular problems or severe cardiovascular or pulmonary conditions. In addition, all were able to walk independently and had no psycho-cognitive dysfunctions in Mini-Cog testing.

### 2.1. Clinical and Neuropsychological Evaluation

Blood pressure, heart rate, and blood oxygen were monitored at rest and after each kinetic session. PD severity was evaluated with the Hoehn and Yahr (HY) scale.

All participants were tested with Mini-Cog for the detection of mild cognitive impairment. None of them had psycho-cognitive dysfunctions and scored 5. All of them had an immediate and short-term memory recall test very good when asked to repeat and remember three randomly chosen words (evaluated 3). The clock drawing test assessing the nondominant hemisphere as well as screening for executive functioning was correct (evaluated 2).

The Mini-Cog test was preferred for its simplicity, rapidity of screening (requiring 2-4 minutes), and because it was free of charge. The sensitivity and specificity of the Mini-Cog are excellent for identifying/excluding early signs of executive and cognitive impairment [30].

Anthropomorphic elements such as body mass and height were collected to calculate the participants' body mass index (BMI, kg/m^2^) (Table 2).

### 2.2. Antiparkinsonian Medications

During the study, the pharmacological treatment was not modified. The majority (15/17 PD) received dopaminergic therapy associated with at least an agonist, except two subjects, who received only dopamine agonists (PR = pramipexole; RA = rasagiline; RO = ropinirole).

Levodopa (L‐dopa) was associated with carbidopa/entacapone (Table 2). Based on theoretical conversion factors, the equivalent daily dose of L‐dopa (LEDD) was calculated for each patient to compare medication regimens. Adding up the LEDDs and dopamine agonist equivalent doses of all the pharmacological drugs leads to a daily total LEDD that is artificial, but feasible and used as a standard computation method [31].

### 2.3. Remotely-Delivered Physical Kinetic Program

Telerehabilitation programs delivered to patients via information technology infrastructure are similar to conventional rehabilitation programs. The physical (kinetic) and occupational therapy were tailored for each patient. For example, during social distancing (October-December 2021), each person attended ten sessions of motor telerehabilitation procedures (2 per week) lasting for 50 minutes each.

The physiotherapy session consisted of 10 minutes of warm-up, toning, and stretching of the lower limbs and trunk axial muscles; 5 minutes of hand tremor control; and 5 minutes to improve breathing. For safety reasons, most of the kinetic procedures were performed in sitting. The remnant 25 minutes were dedicated to endurance, balance in sitting, and orthostatic posture.

Accentuated and rhythmic movements, gait with higher steps, and ample swing of the arms (“*Citius, Fortius, Altius*”), using syncopated, auditory rhythmic cues were aimed at improving walking, balance, and preventing falls.

The physiotherapist's continuous monitoring guaranteed remote supervision and safety. Caregivers/family members were present for technical assistance and patient's safety supervision while performing the exercises.

The physiotherapist provided one-to-one verbal indications and live demonstrations, and coached and corrected possible mistakes during the exercise sequences in real-time.

Sessions were received in the patient's home via a smartphone, computer, or tablet using video meeting systems such as Google Meet, Skype, or WhatsApp.

### 2.4. Evaluation of the Physical (Kinetic) Remote Therapy

“Get up and walk 3 m” (TUG time up-and-go) and “6-Meters Walk” tests summarize some items of the MDS-Unified Parkinson's Disease Rating Scale part III. TUG globally integrates orthostatic balance, control of the essential functional mobility, orientation, and safety of walking dynamics in a functional situation of daily living. Both tests are simple clinical tools used to evaluate the outcomes by comparing results at the initial phase (1) and the end (2) of the rehabilitation program. The two evaluation sessions were carried out on the same day-time, during ON episodes. Three timed examinations were performed, and the results were obtained from the mediation of the last two tests.

### 2.5. Reimbursement

The provider's satisfaction was only moral and professional, without reimbursement for in-home video-based visits (medical services were delivered pro bono, with no professional fee applied nor expectation of remuneration).

## 3. Results

The demographic characteristics, clinical history, anthropomorphic elements, neurological evaluation, and the profile of administered anti-Parkinsonian drugs are summarized in Table 2. The average values, standard deviations, and limits of each item are synthetically mentioned.

Most patients were elderly (limits 54–70 years), with a history of PD between 3 and 12 years. All had a slight or medium functional limitation and were assessed 1.5 to 3 on the HY scale. No patient was mentally disabled. About half of the patients (9/17) were overweight, and 2 (2/17) were moderately obese (class I).

### 3.1. Statistics

*ANOVA* (one-way analysis of variance calculator) and the *t*-test were used to compare the differences between the motor performances registered at the initial (1) and final (2) moments of the study.

### 3.2. Results of the Physical Kinetic Recovery Program

TUG was a global assessment tool for PD patients useful for timing and assessing gait dynamics in a daily functional situation. None of the patients were at increased risk of falling (TUG did not exceed 16 sec).

**Figure 1:**
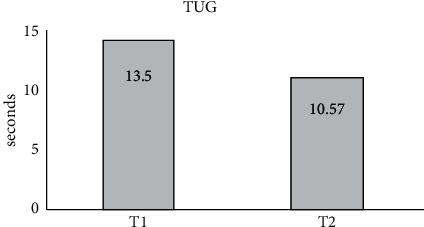
Average TUG before starting the kinetic rehabilitation program (1) and at the end (2).

According to the TUG test evaluation, all patients improved their locomotor performance (Figure 1).

Training significantly reduced the average duration of TUG (from an initial *T*1 = 13.50 seconds, it decreased to 10.57 seconds at T2 (*F* = 6.68, *p* < 0.05)).

**Figure 2:**
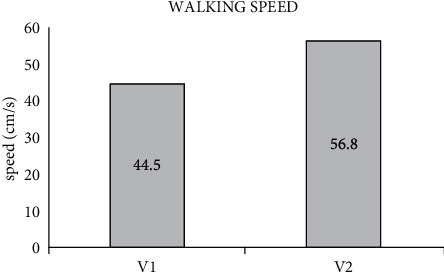
Average walking speed before starting the kinetic rehabilitation program (1) and at the end (2).

Walking speed was significantly improved from an initial average speed of 44.5 cm/sec to 56.8 cm/sec (after kinesiotherapy) (Figure 2) (*F* = 8.1; *p* < 0.05).

There were no significant associations between TUG (or walking speed) and BMI or LEDD.

## 4. Discussion

Abnormal gait patterns (shuffling steps, reduced stride step size, and decreased walking speed) are common neurologic features in people with PD. Walking speed reflects the patient's functional mobility. Deterioration of gait, reduced postural control, bradykinetic movements, and frequent episodes of freezing of gait correlated with the risk of falling [32, 33]. For safety reasons, strict inclusion criteria included the ability to stand up and walk independently for 6 meters, and the mandatory presence of a caregiver or family during the telerehabilitation kinetic session.

The information technology infrastructure allows for evaluating realistic goals and provides the patients with personalized, professional coaching to optimize exercise uptake and adherence to the physiotherapy remote programs. The motor and nonmotor neurological symptoms (speech problems, dysphagia, and cognitive impairments) can be assessed and managed by teleneurorehabilitation [34–40].

The present study used and focused on simple, safe, and easy evaluation instruments, which would hardly give dubious results.

Comparing the interpersonal/office-based assessment method versus home web-based remote evaluation, both seem equally suited to assess PD, primarily because many physical examination findings are visual [39]. However, rigidity and balance quantification are challenging via videoconferencing. Sangarapillai et al. suggested a regression equation that can accurately predict online complete UPDRS-III scores [41].

Telerehabilitation kinetic programs are similar to conventional rehabilitation [42]. Many physical and occupational therapists used health care at a distance to deliver prophylactic education interventions (e.g., fall risk reduction strategies). Stretching and strengthening exercises, coaching to augment functional activities (e.g., transfer maneuvers and stair navigation), and balance training were also addressed [43, 44].

Technology progress can offer the opportunity to safely deliver an integrated healthcare model at a distance and even a simultaneous interaction and training of two or more patients with PD [44, 45].

The client can access and find on the Internet numerous resources and get support from some of the best online therapy services in 2022 [46]. Srivastav and Langer both synthesized comprehensive lists of the iPhone applications indicated for PD patients [47, 48].  Table 3 briefly presents a selective shortlist of applications focusing on PD motor disability and home training.

An advanced PubMed/MEDLINE database search for relevant literature published between 01/01/2020-31/05/2022 used an associative syntax of critical items ((((rehabilitation) AND telemedicine) AND Parkinson) AND Covid) has identified 21 records for screening (titles and abstracts). No filters were added. Fourteen papers in the reference list were relevant for the present study and focused on kinesiotherapy.

The present study has several limitations including the small sample of patients, limited possibilities for a comprehensive clinical examination (e.g., assessment of rigidity and postural instability), and reduced total duration of the program (imposed by the patient's safety and lack of reimbursement).

The group's tiny size was not an isolated problem encountered only in the current study. Garg and Dhamija synthesized a comprehensive list of PubMed-indexed studies concentrating on telerehabilitation in PD and communicated between 2008-2019. Over 60% of the papers related only to small groups of patients (between 8 and 50 subjects) [50].

It is mandatory to adapt telerehabilitation services to the patient's needs and particular socioeconomic conditions in each region [19, 20, 51] There are human, organizational, and technical challenges and also socioeconomic barriers to the emergence of telerehabilitation in different developing countries [28, 52].

Due to the novelty of this method in our department, socioeconomic aspects were considered for this method of evaluation and treatment. Strict criteria for the patients' selection and quality of rehabilitation took into account the access to ICT devices and services appropriate for providing at-home motor telerehabilitation (smartphone, iPhone, tablet, or PC, and Internet access), and the human factor (for technical assistance and safety during the kinetic sessions).

The Internet behavior and the technological limitations were not a barrier to the telerehabilitation of our selected group of PD participants. According to the study “Romanians and the Internet,” conducted by the Romanian Institute for Evaluation and Strategy (IRES), almost two-thirds (64%) of Romanians in urban areas use the Internet, and 71.4% of them access it daily, especially from home. Moreover, 88% of Romanians in urban areas have a mobile phone, and 59% have a subscription [53].

In 2021 the worldwide “digital environment” included 4.66 billion active Internet users (DataReportal, 2021), corresponding to a global Internet penetration rate of approximately 59.5% from 7.83 billion people. Over six out of every ten of the entire world's population had Internet access (Internet World Stats, 2021) [54].

## 5. Conclusion

Despite its human, organizational, technical, and socioeconomic limitations, ICTs applied to medical science might represent a viable solution to countering today's socio-health problems and a realistic opportunity to reduce infection risk. Taking into account the geopolitical particularities and socioeconomic differences between developed and developing countries, telemedicine might offer PD patients coaching, regularity, and continuity of kinesiotherapy, being a convenient remote alternative to the interpersonal, face-to-face model of care [38–40, 55–63].

The information technology infrastructure allows for evaluating realistic goals and provides the patients with personalized, professional coaching to optimize exercise uptake and adherence to the physiotherapy remote programs.

Telehealth can provide flexibility for web-based physiotherapy sessions/programs and offer a holistic picture of the patient integrated into his familiar environment.

The power of telemedicine to validate motor and cognitive clinical examinations and the possibility of remotely supervising, and coaching, PD patients have propelled the telerehabilitation model of interdisciplinary care into the 21st century.

The study confirmed the effectiveness of home-based and remotely supervised physical kinetic rehabilitation (at least for selected cases of PD patients) during the COVID-19 pandemic era, using a tailored program adapted to the individual neurological status in conditions of complete safety. Remote delivery of rehabilitation kinetic programs was appropriate for our carefully selected group of PD patients, although this method was not previously used in our department.

Given the pandemic undulating evolution (with successive waves of exacerbation alternating with periods of pandemic calm down), the hybrid model (associating standard in-person medical assistance with remote-delivered healthcare) might be an appropriate alternative to conventional face-to-face physiotherapy and a flexible model of rehabilitation during the pandemic.

Further studies and ICT programs are necessary to identify the optimal web-based model of care, expand access to video-based care services (remote consultation, patient education, and ongoing monitoring), establish best practices worldwide, and provide equitable access to modern neurorehabilitation [58, 59, 62, 63].

## Figures and Tables

**Table 1:** Participant selection criteria.

Baseline inclusion criteria	Mild or moderately disabling, idiopathic PD (score range 1.5-3 on HY scale, in “OFF” state) No history of positive COVID-19, nor recent contact with positive people Not currently receiving physical therapy or occupational therapy Able to stand up and walk independently 6 meters Able to follow verbal commands Able to provide informed consent (agreement to be filmed and photographed while maintaining the elements of confidentiality) Stable pharmacological treatment for the last six months Access to ICT devices and services appropriate for providing at-home motor telerehabilitation: smartphone, iPhone, tablet, or PC, Internet access Personal automatic digital sphygmomanometer and a pulse oximeter (a few subjects had a smartwatch) The caregiver's presence and family mediation were mandatory (for technical assistance and safety during the telerehabilitation kinetic session)

Exclusion criteria to maintain safety	Age over 70 Severe comorbidities: History of cardiac conditions (myocardial infarction, uncontrolled arrhythmias, and congestive heart failure 3-4 NYHA) History of chronic obstructive pulmonary disease (COPD) and use of supplemental oxygen History of stroke, cerebral tumor, and severe traumatic brain injury Orthostatic hypotension (systolic BP < 110 mmHg) or uncontrolled resting hypertension (systolic BP > 180 mmHg or diastolic >110 mmHg) Uncontrolled diabetes mellitus Disabling arthritis or severe pain (that could limit physical activity) Visual and auditory impairments that disrupt audio-video interactions Cognitive impairments (dementia and aphasia) that prevent the patient from understanding audio-video information and signing the ethical consent form. Patients quantified ≤3 with the Mini-Cog test History of same-level falls occurred in the last six months DBS (deep brain stimulation) or continuous duodenal levodopa infusion (levodopa/carbidopa intestinal gel)

**Table 2:** Clinical characteristics and profile of administered anti-Parkinsonian drugs.

N	Gender	Age	Weight (kg)	Height (cm)	BMI	PD years	HY	Mini-Cog	Medication (mg/day)	LEDD (mg)
1	M	70	76	160	29.7 (ow)	12	3	5	LD (750) PR(2.1) RA (1)	1110
2	M	63	75	160	29.3 (ow)	4	2	5	LD (600) RA (1)	700
3	M	70	63	167	22.6	6	2	5	LD (750) PR (0.18) RA (1)	868
4	F	68	68	168	24.4	11	2.5	5	LD (800) RA (1) RO (4)	980
5	F	58	67	167	24	11	2	5	LD (800) PR (1.06)	906
6	M	66	69	166	23.1	3	2.5	5	LD (475) RO (24)	955
7	M	66	65	172	22	6	2.5	5	LD (600) RA (1) RO (4)	555
8	M	70	70	165	25.7 (ow)	12	2.5	5	LD (600) PR (2.1) RA (1)	910
9	F	59	90	173	30.1 (mo, cl-1)	8	2	5	LD (600) PR (2.1) RA (1)	910
10	M	70	81	180	25 (ow)	9	2.5	5	LD (600) PR(0.52) RA(1) AM (100)	852
11	M	54	68	167	24.4	7	2.5	5	RA (1) RO (16)	420
12	F	70	68	160	26.6 (ow)	2	1.5	5	PR (0.52)	52
13	M	69	75	170	26.0 (ow)	5	2	5	LD (200) RA (1) RO (8)	460
14	F	67	75	152	25.4 (ow)	10	2.5	5	LD (800) PR (0.26) RA (1) AM (200)	1126
15	M	67	81	169	25.4 (ow)	3	2	5	LD (500) PR (1.58)	658
16	M	64	94	190	26.0 (ow)	3	2.5	5	LD (800) PR (0.52)	852
17	M	70	92	172	31.1 (mo, cl-1)	12	2.5	5	LD (800) RA (1)	900
Average		65.9 ± 4.87 years	75.1 (±9.6) kg	168.2 (±8.5) cm	25.92 (±2.6)	7.3 ± 3.6 years	2.3 ± 0.35	5	—	777 (±275) mg
Limits		54–70 years	65–92 kg	152–190 cm	22–31.1	3–12 years	1.5–3		—	52–1126 mg

HY = Hoehn and Yahr stage; AM = amantadine; LD = levodopa; PR = pramipexole; RA = rasagiline; RO = ropinirole; LEDD = L-dopa equivalent daily dose (mg/day); ow = overweight; mo, cl 1 = moderate obesity, class I.

**Table 3:** Smartphone-based applications used in virtual reality-based rehabilitation of gait and balance in PD (modified from [47–49]).

E-Rehab apps	Aim
PD Warrior https://play.google.com/store/apps/details?id=com.pd.warrior&hl=en_IN	Daily kinetic exercises to improve physical activity
ListenMee https://play.google.com/store/apps/details?id=com.brainmee.listenmee&hl=en_IN	Training of the gait parameters (cadence, stride length, and walking speed) by providing auditory queuing
Parkinson mPower study app https://apps.apple.com/us/app/parkinson-mpower-2/id1375781575	Monitoring and managing the cardinal motor symptoms of PD (gait, balance, and tremor)
Parkinson Home exercise https://apps.apple.com/us/app/parkinson-home-exercises/id473641730	Home-based physiotherapy exercises/programs to improve the balance, gait, and daily living activities
KinesiaU https://www.glneurotech.com/products/kinesiau/	Low-cost consumer app using iPhone or Android smartphone and smartwatch, for motor assessment in PD

## Data Availability

All data are included in the manuscript.
